# Navigating power hierarchies in the “field”: A qualitative exploration of researcher experiences

**DOI:** 10.1371/journal.pgph.0006048

**Published:** 2026-03-11

**Authors:** Michelle Lokot, Agata Pacho, Thurayya Zreik, Rahma Hassan, Nada Abdelmagid, Mishal Khan

**Affiliations:** 1 London School of Hygiene and Tropical Medicine, London, United Kingdom; 2 Purdue University, West Lafayette, West Lafayette, United States; 3 Independent Consultant, Beirut, Lebanon; 4 Institute of Development Studies, University of Nairobi, Nairobi, Kenya; Institute of Tropical Medicine: Instituut voor Tropische Geneeskunde, BELGIUM

## Abstract

Despite increasing focus on the broad topic of power hierarchies within research partnerships, scholarly analysis and guidance within global health tends to focus on partnership-building, governance structures and authorship, while the interactions occurring between researchers in settings where a project or community are located, often referred to as the “field”, remains a neglected topic. This study applies an intersectional lens to explore how to tackle power hierarchies (including race, gender, age, education/expertise) during visits in the “Global South”, based on a case study of an academic institution in the United Kingdom. It involves interviews with staff and research degree students, as well as research partners, and feedback workshops. The study finds that positionality influences how participants view the role of power hierarchies in shaping research dynamics. Senior staff tended to be less critical of power hierarchies, while early-career researchers were more inclined to feel power hierarchies needed to be challenged. Across multiple types of research relationships we find that seniority is a powerful dynamic that shapes interactions during visits. Institutional power is also an overarching force that often limits individual researcher efforts to shift power. Our study identifies five key recommendations for Northern-based institutions in particular: 1) challenge extractive practices and assumptions associated with research in the “field”; 2) advocate for more equitable institutional policies and practices on contracting and budgeting in Northern institutions that constrain efforts to shift power; 3) build in time for ongoing reflection on power and positionality within research teams; 4) ensure visits to “the field” are planned with Global South partners; 5) conduct further research on power hierarchies to tackle specific dimensions of power.

## Introduction

Power hierarchies are inevitably present in any research partnership. Researchers working in disciplines and fields such as anthropology, sociology, gender studies and post-colonial theory have grappled with power hierarchies within research for decades, advocating for challenging hierarchical research practice. This includes proposing decolonial research methodologies that are more centered on indigenous ways of knowing [1], challenging how different forms of knowledge are valued over others [2], seeking to ground research in relationality to challenge the idea that research is objective [3], and shifting from essentialist descriptions of participants’ lives to context-specific analysis [4,5]. Other key contributions that have been instrumental in shifting thinking on how research is conducted include approaches like community-based participatory research, participatory action research, and creative and arts-based research, all of which are underpinned by an understanding of how power hierarchies shape research [6,7].

Within global health, momentum towards considering power hierarchies in research has been relatively recent and has tended to focus on shifting governance structures, redistributing resources, challenging assumptions about capacity, promoting equitable authorship and encouraging shared decision-making in research partnerships [8–12]. Narratives about decolonising research and shifting power in global health do not tend to focus on how to approach power hierarchies during visits to “the field” – which, despite the critique of the term, continues to be a commonly used term. These in-person interactions in settings closer to where a project/community are located can involve activities such as attending meetings, participating in training, identifying research partners, strengthening research partnerships, designing studies, and collecting, analysing and sharing data. Each of these direct interactions create unique and fluid power dynamics. In the context of this research study, travelling to “the field” relates to researchers from a UK-based university travelling to other settings in the so-called “Global South” and the resulting power hierarchies from such in-person interactions. Specifically, in this paper, we use an intersectional lens to explore how to tackle power hierarchies (including race, gender, age, education/expertise) during field visits in the Global South, based on a case study of an academic institution in the United Kingdom: the London School of Hygiene and Tropical Medicine (LSHTM). Founded as part of the British colonial empire [13], LSHTM is one of the leading public health research institutions globally, and its staff conduct research in over 100 countries.

Notwithstanding the geographical inaccuracies associated with dividing the world based on “North” and “South” [9], knowledge and capacity are often framed as flowing from North to South, such that researchers in the North might travel to the South to share their expertise. Similarly, while we focus on the field of “global health”, we remain aware that the discipline is an “invention of a largely white and wealthy elite residing in high-income, English-language speaking countries” [14] with roots traceable to colonialism. Consequently, the identification of global health problems in the South, as well as the proposed solutions, tends to be determined by this privileged group [15].

### How we understand power

The concept of power hierarchies broadly refers to systems (such as gender, race, class, age, education, expertise, colonialism – among others) that shape how people are organised and that affect dynamics between them. There is a significant body of scholarship on the concept and theory of power. Foucault framed power as something which “circulates” and “functions in the form of a chain” rather than being “localised” or fixed [16], as well as being linked to space and place [17]. Feminist scholar Kimberlé Crenshaw importantly argued that analysing individual power hierarchies in isolation is insufficient and that understanding power requires recognising how different power hierarchies intersect to complicate single-issue understandings of lived experience [18]. Understanding the complex ways in which power is enacted is also part of Patricia Hill Collins’ “matrix of domination” framework. She has suggested that power is exercised and reinforced within four domains: structural (within systems, laws and institutions), disciplinary (involving how individuals are controlled within these structures), hegemonic (referring to the norms and ideologies that reinforce and justify oppression) and interpersonal (involving the everyday relationships and interactions that reinforce power) [19]. Her framework has been used to identify the power hierarchies present in global health [8]. These frameworks for identifying and understanding power call attention to how hidden and direct forms of power operate in interpersonal relationships, institutions and society more broadly. Within global health research and academia more broadly, some systems of power are more pervasive than others.

### Critiques of power hierarchies in global health

Efforts to tackle colonial power hierarchies present in knowledge production and academia have been informed by activists in the Global South, and are understood as essential to the broader project of decolonising land, institutions, and social systems [1,20,21]. Aníbal Quijano has emphasised that colonialism is inspired and preserved by ensuring knowledge production meets the needs of capitalism, including measurement, quantification and the idea of objective knowledge. This dominant way of knowing the world continues to shape how knowledge is viewed and extracted, including within research [21]. Further, since the killing of George Floyd and the Black Lives Matter protests in 2020, there has been greater attention on power hierarchies including race, imperialism and colonialism [22]. More attention has been given to the dynamics of neocolonialism, which enable the former colonial powers to exert indirect influence over previously colonised countries [23]. Global health scholars and practitioners have urged the importance of critically reflecting on their power and privilege and taking steps to tackle unequal power hierarchies [24–28]. For global health research, this has meant a focus on decolonising research methods, promoting equitable research and encouraging approaches like co-production, focusing on dynamics between research partners in the “North” and “South” and interactions with research participants [11,25,29,30]. However, existing guidance on power hierarchies tends to focus on partnership-building, governance structures that enable power-sharing, shifting decision-making partnerships and promoting equitable authorship practices, while dynamics during in-person engagement in “the field” remains a neglected area.

### Power hierarchies in “the field”

While global health scholarship has not sufficiently examined the dynamics that unfold in “the field,” we draw on disciplines that have paid closer attention to field engagement within research partnerships, such as anthropology, to explore how these issues have been addressed. Long regarded as a “rite of passage” [31], an indicator of credibility for researchers, demonstrating their expertise, “mastery” and deep knowledge of a context [32,33], “fieldwork” has been interrogated for its links to colonialism [31], privileging certain forms of knowledge over others and creating a false dichotomy between being “home” and “abroad” [34,35].

Scholars have argued that attention to researchers’ “embodiment” in the field, shaped by the intersecting power hierarchies they inhabit, often remains superficial, and that merely acknowledging these identities is insufficient [36], “as if the body, once acknowledged, can then be written out of the work” [37]. Such obscuring of power hierarchies occupied by researchers can mean a “sanitizing” of research experiences [38]. They note that fieldwork tends to be viewed as masculine due to gendered power hierarchies in academia [36,38,39], which has resulted in female researchers feeling they cannot raise issues related to sexual harassment and other forms of gendered violence during fieldwork because of risking being seen as unprofessional, unqualified or emotional [31,36,39]. Race has also been a power hierarchy identified as shaping research experiences, and which intersects with gender during research processes [40,41]. On the other hand, “whiteness” and “foreigner” status may give certain researchers power and privilege [42,43].

However, there is also growing critique about assumptions made about “insider” and “outsider” positions during research, because these may be blurred or can shift in certain spaces [44,45]. Recognising positionality as something involving “subtle shifts” along a “spectrum” is therefore key, alongside asking questions like: “Why does the researcher’s assumed ethnic belonging become more significant in some situations than others?” [44]. Indeed, the researcher’s power cannot be taken for granted - at times their power is limited [46]. Researchers also make strategic choices to present themselves in certain ways, for example positioning themselves as “learners” or displaying “performed approachability” to be more accepted [45]. At times, researchers may find themselves subject to the decision-making of others, such as gatekeepers who, while instrumental in facilitating access, can also slow, restrict or even manipulate research processes [47]. Further complexities can occur when translators or research assistants are involved in research [48].

Within the discipline of anthropology in particular, there is a growing discussion about what may help researchers process and navigate power hierarchies during fieldwork. These include strategies that are grounded in collaboration and interdependency [49] to help combat the typical isolation researchers experience, such as group journaling and debriefing with translators/research assistants [47]. Within such collaboration efforts, Nagar stresses the importance of scholars engaging in “radical vulnerability” that invites critique and assessment from others [50]. From anthropology has also emerged the idea that data collection should only occur after a researcher has been immersed in the context for some time and has deeper knowledge of the setting [51]. Within the literature, there is clear recognition that researchers are not always sufficiently prepared to navigate complex power hierarchies present during fieldwork. This includes being uncertain of how to respond to gender-based violence and insecurity [52].

### Our aim and objectives

Informed by the critique of power relations in “the field” identified by other scholars and disciplines, this research sought to apply an intersectional lens to explore how to tackle power hierarchies (including race, gender, age, education/expertise) during field visits in the Global South, based on a case study of the LSHTM. The three research objectives were to: 1) Explore staff, Research Degree student and partner perspectives on power hierarchies and the value of “field” visits; 2) Identify how power hierarchies may influence interactions during field visits, particularly between LSHTM staff and local research collaborators; and 3) Develop key guidance for LSHTM staff to navigate and address power hierarchies during field visits, considering gender, anti-racism, decolonisation, cultural sensitivity and micro-aggressions.

## Methods

### Ethics statement

This study received ethics approval from the London School of Hygiene and Tropical Medicine Ethics Committee (ref: 30703, 29^th^ May 2024). Data collection occurred from 3^rd^ June 2024–31^st^ January 2025. All research participants were given a Participant Information Sheet and provided written consent.

### Research participants

Overall, 36 individuals participated in this study. This includes 20 interview participants and 16 participants who only attended feedback sessions. All data collection occurred using Zoom.

The 20 interview participants consisted of: 9 LSHTM staff members, 5 Research Degree students undertaking doctoral research, and 6 individuals from organisations in the Global South who previously or currently had research partnerships with LSHTM. LSHTM participants were invited to participate through open recruitment calls advertised in internal newsletters and mailing lists and direct emails. LSHTM participants were encouraged to suggest partners who might be interested in participating and these individuals were invited by email.

### Data collection and analysis

Interviews took approximately 45 minutes. Interviews were conducted by ML and were audio recorded and transcribed using Zoom. During interviews, participants were asked how they would like to be described with respect to gender, racial/ethnic identity and seniority (early-career/junior, mid-career or senior) to help nuance the analysis. ML took detailed notes of observations and key themes from each interview, which formed the basis of the inductive coding approach that was taken. Codes were identified from interview themes and Nvivo 12 software was used to inductively code transcripts. Codes were refined during the coding process and grouped thematically to identify the higher level themes that helped to structure the writing.

Following analysis, all interview participants and any other interested LSHTM participants or partners were invited to attend feedback sessions, which were designed to provide an opportunity to hear initial findings and recommendations and provide further input. All additional participants provided written informed consent. In total, 3 feedback sessions were held with LSHTM participants and 2 were held with partners; these were attended by 26 individuals. This included 10 interview participants and 16 additional participants; in total 20 feedback session participants were from LSHTM and 6 were participants from partner organisations. Feedback sessions ran for approximately 1.5 hours and were facilitated by ML, who took notes on participant contributions and integrated these into the analysis.

### Positionality of the authorship team

The authorship team consists of the lead researcher (ML) and an Advisory Group who provided oversight and guidance during key points of the study, including research design, analysis and writing (AP, TZ, RH, NA, MK). The authors are a mix of LSHTM staff (ML, AP, NA, MK) and LSHTM previous/current partners who are based in the Global South (TZ, RH), all of whom have been involved in research. Each author has been involved in challenging research power hierarchies within LSHTM and beyond. Many of the authors identify as women of colour. The authors have experienced the complex dynamics of how gender, education level, skin colour, class, age, seniority, nationality, economic status and institutional affiliation intersect to at times privilege and other times disadvantage them in their roles as researchers. Their own experiences have undoubtedly informed and strengthened the research design and analysis process. Individual positionality statements of the authors are provided in our S1 File.

## Findings

The findings consist of two main sections. Firstly, we outline how power is understood by participants. Secondly, we describe how power dynamics were experienced and observed by participants. This second section breaks down dynamics into four types of relationships to explore the multi-layered interpersonal and institutional dynamics that shape research: a) LSHTM-LSHTM dynamics, b) LSHTM-partner dynamics, c) Dynamics within partner organisations; and d) Dynamics with research participants and other stakeholders.

### Power: how participants think about it

For some participants, it seemed easier to think about what power hierarchies are and how they impact research processes. Power hierarchies were viewed as self-reproducing, inevitable, and evolving over the course of a person’s life. One participant described power as “like a whole maze of organic relationships that keep reproducing themselves” from the lowest to the highest level (Interview 4, woman, mid/senior, partner). For another, power hierarchies were an inevitable part of life: “there’ll always be power hierarchies”, but “in an ideal world” these might have less influence (Interview 12, woman, mid-career, LSHTM). Participants described various forms of power hierarchies they had experienced and observed in the “field”, including seniority, age, class, professional hierarchies (such as having medical qualifications, or being a researcher holding more power than a practitioner) and race. For those in more senior roles, there was some reflection about how power hierarchies occupied by an individual would change over time: “[M]y age has changed so… my professional seniority has changed. So my power vis-à-vis other people will have changed’ and also depended on context (Interview 2, woman, senior, LSHTM). However, for others, there was discussion about how talking about power was not necessarily easy: “some of the language around power dynamics is perhaps not easily very clear, or easily understood” (Interview 18, man, senior, LSHTM).

We found that individual positionality may have played a role in shaping perspectives about power. Those who were more senior LSHTM staff and who identified as White were less likely to be as critical of power hierarchies. This included suggesting that power dynamics “may not necessarily be harmful in any way” (Interview 18, man, senior, LSHTM). One participant emphasised that it is not always straight-forward to identify who has power and who doesn’t, suggesting it was problematic to assume that LSHTM actors always hold power: “I think sometimes we’re a little self-important, like, oh, we’re so amazing. We must just like wreak power dynamics all over the world… And actually, people are not that fussed about white academics” (Interview 2, woman, senior, LSHTM). Another participant seemed to indicate they had faced negative impacts from being assumed to have power: “[I]t’s hard to apologise for being privileged. And so I think the idea should be that you use your privilege for good to empower other people… you can help lift up their voice and… put them in positions of power” (Interview 20, woman, senior, LSHTM). During a feedback session, there was also discussion about not “scaring people off travel”, with concern expressed by a few senior staff that early career staff in particular have a perception that travelling to the Global South is difficult and people need to be careful when engaging with other cultures. This concern that research travel is being over-complicated by conversations about power may also be indicative of positionality.

However, early-career participants at LSHTM tended to have more negative perceptions of power hierarchies within research, being stronger in expressing the view that being White and/or being associated with LSHTM conferred power on researchers. Early career participants pushed back on the idea that senior White researchers who had been working in the same country/region for a longer period were not really outsiders anymore because they knew the setting well, emphasising that power hierarchies were still present in these situations. Early career staff also expressed concern about power hierarchies not being addressed by senior researchers: “[I]t’s only the kind of younger early-career researchers, and then a few senior people that seem to be kind of have this mindset [to challenge power]”, commenting that academia was an “unfair system” where early-career researchers had less say and freedom to run projects more equitably, which would lead to this group of younger researchers leaving academia “because we can’t really challenge the system enough”. She observed: “And then all that will be left is the ones who don’t actually care about it” (Interview 1, woman, early-career, LSHTM).

Participants also considered the relationship between individual positioning within power hierarchies and the capacity of researchers to exercise agency in challenging those structures. There was a very strong sentiment by LSHTM participants that the institution of LSHTM holds significant power despite the efforts of individuals: “because of the structures with the donor and finances… stuff that was out of our control” (Interview 12, woman, mid-career, LSHTM). Interestingly, one participant from a partner organisation suggested that not everything was about LSHTM having power, but individual personalities also affected interactions: “[I]nitially, it felt like it was institutional, Global North, Global South. Now I feel it’s more based on the person you’re interacting with at the London school” (Interview 16, woman, mid/senior, partner). In one feedback session, there was also discussion of the difference between culture and power.

Participants also reflected that sometimes LSHTM was less hierarchical and “traditional” than “academia in some countries” (Interview 13, woman, mid-career, LSHTM). There was recognition that power is talked about more and that there is greater appetite to challenge unequal power hierarchies within research: “[T]here has been a move in the right direction where people around the world know that they can tell us to shove it if we start acting a little bit above our stations”, linked to both increased access to higher education in many countries and “more introspection” among Northern researchers with respect to power (Interview 2, woman, senior, LSHTM).

### Power dynamics across different relationships

#### LSHTM-LSHTM dynamics.

This section discusses insights about the dynamics between different LSHTM actors who travel together, recognising how dynamics between LSHTM staff also shape other relationships, including those with partners. Academic seniority emerged as a key dynamic affecting relationships during visits. Additionally, gender, race and immigration status (and the intersects between these) were discussed by participants.

**Seniority:** Across interviews and feedback sessions, seniority was overwhelmingly what LSHTM actors most discussed when talking about LSHTM-LSHTM power dynamics. Participants described experiences having to cover for senior staff who were unprepared, having to redo the work of senior staff without recognition, being required to have early morning meetings with senior staff without an apparent reason, and even supporting senior staff with more menial tasks: “I’ve worked with [Principal Investigators], who are… like you are literally getting coffee for them” (Interview 12, woman, mid-career, LSHTM). LSHTM actors described how senior staff would exclude them from the politics of projects, saying things like, “Oh, you don’t have to worry about that kind of thing like that…it’s between us like more senior people”, which left early-career staff feeling uncertain: “It just added another layer of complexity in terms of me trying myself to navigate those hierarchies there… it was very much kind of under some sort of veil that I couldn’t like see through” (Interview 15, woman, early-career, LSHTM). Research degree students who were travelling for data collection also felt less confident asking supervisors for help: “You feel so awkward emailing your supervisors like once a month with a question” (Interview 1, woman, early-career, LSHTM). Importantly, there were a few examples mentioned of positive interactions with senior staff, with early-career staff and students feeling their opinions were valued and hierarchies were less evident.

**Gender, race and immigration status:** Gender hierarchies among LSHTM staff presented through women being placed in organisational roles for visits, for example being responsible for corralling people, arranging the room for a meeting, or taking notes. Senior male LSHTM actors were recognised as having specific privileges, but there was also discussion of them using their privilege to “open doors” for more junior female staff (Interview 2, woman, senior, LSHTM). Race and immigration status were also discussed as intersecting with gender, with female staff who were dependent on LSHTM for visas and who held precarious contracts feeling they needed to over-perform or “volunteer” for additional tasks during a trip: “I need to almost prove myself… so that when my contract comes up for renewal my boss or the PI will fight for me so that I can get another… There is a sort of like need to perform, and maybe sometimes over-perform, especially if you feel that your contract is precarious” (Interview 12, woman, mid-career, LSHTM). Immigration status, specifically not having a UK passport, was also discussed as creating challenges and power dynamics that were not always well-understood by LSHTM staff: “I feel sometimes that the LSHTM doesn’t have an idea how traveling with a non-British passport looks like at all at all… their ability to kind of even solve any of these issues that is relevant to visa travel for Global South researchers, they are absolutely in a different place” (Interview 10, woman, early-career, LSHTM).

#### LSHTM-partner dynamics.

Within the LSHTM-partner relationship, key findings related to the role of seniority in shaping relationships. Other issues discussed include the influence of gender, race and ethnicity, and the overarching institutional power of LSHTM. Assumptions made by LSHTM actors and examples of poor LSHTM behaviour during visits are also discussed by participants, along with examples where LSHTM actors felt they had less power. We discuss key tensions and challenges associated with shifting power and end this section by discussing the timing, scope and value of in-person visits.

**Seniority:** The issue of seniority between LSHTM and partner actors was often discussed as a key power hierarchy affecting interactions. A senior LSHTM participant suggested that being more senior “can make you less likely to throw your weight around because you’re not proving anything anymore” compared to someone who was early-career (Interview 2, woman, senior, LSHTM). She contrasted pressure on early-career staff to prove themselves with someone more senior who knew which battles to fight: “[W]hen you get more experienced you don’t care so much that it’s not being done exactly the right way”. Other senior staff also reflected on how their seniority meant partners were less likely to challenge them: “[A]s you become more senior, you do wonder whether you’re not challenged as much as you maybe were when you were more junior… as you become more senior… you can often get your way” (Interview 18, man, senior, LSHTM). LSHTM actors discussed the tension between being seen as too soft or junior versus as someone who was taking over the project.

LSHTM participants who were early-career found it easier to engage with partner staff who were also at a similar level: “the other local staff I think were a bit intimidated by my supervisors… because it was more senior people that maybe the dynamic changed slightly” (Interview 1, woman, early-career). Partner staff also found it easier engaging with early-career LSHTM actors:

“The senior people… the way we relate is different ‘cause on one hand, we hope they like us and keep working with us. The junior ones, it’s more flexible. We can get them involved in a lot of work, in the day-to-day smaller tasks… For the senior ones, the relationship is different, how we interact it’s naturally different cause the scale is already tipped… It’s hard to say… Can you do this? It’s hard” (Interview 4, woman, mid/senior, partner).

Despite this preference for early-career actors to engage with their early-career counterparts, at times LSHTM participants felt that their junior status meant they could not directly ask a partner something but a senior colleague had to be the one asking.

However, there was also a sense that sometimes if LSHTM actors were too young, partners might view this as “disrespectful”. One early-career LSHTM actor was told by a senior LSHTM actor, “No one will respect you until you’re my age” (Interview 15, woman, early-career, LSHTM), suggesting respect is gained over time, rather than being automatic. On the other hand, partners expressed the perception that LSHTM actors automatically had prestige and knowledge:

“We believe that the Western people, that Western academics are the expert in any field… we think that they are the correct anytime… [W]e are not happy to provide our actual ideas and views… [in] our language [it’s] very difficult to say our ideas” (Interview 19, man, mid/senior, partner).

LSHTM actors expressed discomfort with being positioned as experts by default: “They [Global South partners] were very much taking… a back seat in those discussions that made me feel kind of uneasy” (Interview 15, woman, early-career).

**Gender, race and ethnicity:** LSHTM participants who were women described feeling pressure to adhere to stereotypical expectations of how women should behave in particular settings: “not to come across a strident or aggressive, or things that I would have thought were just assertive,” including “learn[ing] the rules of the game… play[ing] by those rules” (Interview 2, woman, senior, LSHTM). Partners were less likely to feel gender was an issue in interactions with LSHTM actors, with some recognition also of the positive role of “really senior women leading projects” (Interview 4, woman, mid/senior, partner).

Race and ethnicity were also discussed as influencing the power of LSHTM actors. For example, being seen as a “foreigner” gave an LSHTM woman researcher more power than partners who were women. However, some LSHTM participants also expressed frustration with being assumed to have privilege or power because of having white skin colour, and not being seen in more complex ways outside of race because of their lived experience.

**LSHTM’s institutional power:** LSHTM’s institutional power and frequent role as budget holder was discussed as having a powerful impact on dynamics between LSHTM staff and partners. While one partner indicated that they felt LSHTM staff did not “show any sort of hierarchy”, they also reflected on how money affects the dynamic with LSHTM:

“[T]here’s a feeling inside that yes, you have the money… you don’t feel that you’re 100% relaxed…You feel that if you did, if you do any mistake that could affect this relationship… it’s not a hundred percent equal partnership…because it’s their money. They hold the money. And you actually are the weaker, partner in this relationship” (Interview 11, man, mid/senior, partner).

LSHTM participants also expressed disappointment with the institution’s bureaucracy and administrative processes, which they felt represented LSHTM exercising power over partners. Slowness in signing contracts, insurance requirements and contracting processes were described as “unreasonable” and “embarrassing” (Interview 13, woman, mid-career, LSHTM). In a feedback session, one LSHTM staff member described being “ashamed” and having to “apologise for the School” when basic processes were not in place to facilitate the research partnership. Another LSHTM actor commented on LSHTM’s accounting rules: “They couldn’t receive any money unless they jumped through the hoops that we had set before them” (Interview 3, man, early-career, LSHTM). At times, donors reinforced LSHTM’s institutional power, rendering the efforts of individuals less effective:

“As much as we’re enabling or saying to the countries, the country partners, yeah, you guys do this. The donors still come to us… So there’s technically, they were in charge. But they weren’t in charge management wise just because of the way the structures are set up of grants and donor reporting and finances... So again, you know, quite, quite uneven” (Interview 12, woman, mid-career, LSHTM).

However, there was also recognition that globally, these dynamics were shifting and LSHTM’s role as budget holder needed to be reconsidered because of donors shifting to giving funds to local actors directly: “We’re probably not going to be running the projects anymore” (Interview 13, woman, mid-career, LSHTM).

The overarching power of LSHTM as an institution could account for some of the responses from participants that indicated that partners do not tend to push back on what LSHTM actors suggest, or take opportunities provided by LSHTM actors for them to lead:

“[E]ven when we would try and turn things back, turn things around and try and make things more of an equal or discussion amongst equals… the conversations or the direction of travel always went towards that kind of ingrained hierarchy” (Interview 2, woman, mid-career, LSHTM).

LSHTM participants shared examples of partners saying “yes” when they meant “no”, partners tolerating poor behaviour of LSHTM staff and feeling intimidated by senior LSHTM staff. Partners also indicated they felt discouraged to ask questions because of being shy and also felt language was a barrier to being able to actively contribute their perspectives.

Importantly, a few LSHTM actors challenged the common perception that partners always had less power than LSHTM because of the power of the institution, sharing examples of partnerships where this was not the case. One early-career staff member shared the example of being “ghosted” by a partner, who, perhaps not wanting to collaborate, avoided any contact and stopped responding to emails (Interview 8, woman, early-career, LSHTM).

#### Problematic behaviour of LSHTM actors.

Participants identified instances of problematic behaviour by LSHTM researchers, some of which, given the institution’s position of power, could be understood as abuses of that power. These behaviours may also be linked to seniority. Examples of poor behaviour mentioned by partners and LSHTM actors included LSHTM actors badmouthing each other to the partner, asking partners to use their networks and then not acknowledging their efforts, allocating their work without discussion when the partner went on maternity leave, being disrespectful to partners, and raising their voice towards partners. Early career LSHTM participants described having to explain the poor behaviour of senior LSHTM staff to partners. They also felt unable to take action because of their positioning in the LSHTM hierarchy: “my ability to do anything about it was really compromised by where I sit in the London School hierarchy” (Interview 9, man, early-career, LSHTM).

A key area where power hierarchies remained in favour of LSHTM and resulted in problematic behaviour from LSHTM related to the timing of LSHTM visits, which would often occur on LSHTM terms, requiring partners to “make time” and “to align, because many times they are the grant owners… so we have to fall in place” (Interview 4, woman, mid/senior, partner). These visits meant partners would “put aside all [their] other work” to host the visitors, however partners felt the same might not be true if they visited the UK (Interview 5, woman, mid/senior, partner). Partners indicated that LSHTM actors seemed unaware of public holidays in their setting yet expected partners to know about Christmas breaks and their office closures over holiday periods. LSHTM actors often scheduled visits during the summer but without considering partner timings: “Just because it’s summer for you… doesn’t mean I’m free” (Interview 4, woman, mid/senior partner). For some partners, hosting LSHTM had consequences for their weekends and vacation times, but they felt unable to communicate this: “[I]t’s difficult to me say… this is my vacation… usually we don’t express that publicly or loudly. We keep the work without… objecting” (Interview 11, man, mid/senior, partner). In a feedback session, one partner participant mentioned being called back from annual leave due to a last minute LSHTM visit. LSHTM participants recognised the efforts partners made to host them, recognising partner visits may not be treated the same way: “I feel like people are much more gracious hosts when we go to a country than we ever are when they come to London” (Interview 20, woman, senior, LSHTM). LSHTM participants expressed their guilt at taking up partner time due to their visits, for example sharing how the visit heightened security risks for partners due to needing to transport LSHTM staff to data collection sites.

Partners also indicated that the purpose of LSHTM visits was not always clear. Partners did not always have a say in the agenda of the visit, with one partner sharing an example of an LSHTM actor working from their office without anyone knowing the reason for their visit. In a feedback session, one partner reflected, “We think they know it all and are coming to see our mistakes”. However, there were also examples of more joint planning of visits, including efforts to “collaboratively developed a schedule” and discuss “what we want to accomplish like really meaningfully” (Interview 7, woman, mid/senior, partner). Partners indicated that LSHTM actors could be more aware of the context and partner ways of working. A need to be aware of university structures in different countries and pressure to teach, which can lessen time for research, was also discussed as important. Partners also expressed concerns with new collaborations being sought during humanitarian crises from researchers who did not know the setting, suggesting it was important for LSHTM actors to have experience working in settings before commencing research collaborations.

LSHTM participants described dynamics that could be understood as partner reactions to previous poor behaviour of LSHTM actors. These included examples where partners had previous negative experiences with other universities or with LSHTM specifically and therefore entered new partnerships with LSHTM with a very strong desire to prevent these dynamics in future:

“And instead of a warm welcome I get… People in your organisation have really screwed us over multiple times… so kind of just making it really clear… We will not stand for this again, like we are going to be senior author on everything, perhaps even first author on stuff… [W]e [LSHTM] pretty much ceded power… because they felt, really, you know, hard done by London School and a number of other projects, but almost in a way where I felt like we were paying the price for someone else’s… mistakes” (Interview 12, woman, mid-career, LSHTM).

Many participants discussed how, despite these challenges, in-person interactions between LSHTM and partners were important for research, helping with understanding the constraints of research settings: “I generally think people don’t do enough going out… [I]t’s worse to be doing [this work] where you don’t understand that where these people live the power goes out, and they have to spend half a day a week just sourcing water for the rest of the week… it doesn’t help to have more distance” (Interview 2, woman, mid-career, LSHTM). Some LSHTM participants also questioned the need for LSHTM presence, feeling it was not always necessary or could be misinterpreted. Socialising informally outside the work setting during these in-person trips was consistently discussed as vital to strengthening relationships between LSHTM and partners. These experiences of “social bonding” were described as “crucial” to research partnerships (Interview 1, woman, early-career, LSHTM).

**Tensions and challenges in shifting power to partners:** LSHTM actors found it challenging to identify how to shift power to partners while at the same time use their skills and experience, feeling there was a tension between shifting power and ensuring what they understood to be standards of robust research. They described not wanting to “pull rank” or be seen as “aggressive” but also still give feedback (Interview 14, woman, mid-career, LSHTM). A few indicated feeling uncomfortable to be seen as supervising or assessing the work of partners, while others specifically held back their views due to a “subconscious feeling of not being able to just say and contribute what you think” (Interview 15, woman, early-career, LSHTM). LSHTM actors shared their worries about being called “colonial” and felt this fear motivated silence on some issues. There was also discussion about when LSHTM actors should intervene if they observed problems, such as poor treatment of other local staff, data collection teams or drivers. Evaluations were discussed as an area of work where LSHTM participants felt particularly uncomfortable because their role was to assess an intervention, but they also wanted to have positive relationships with partners.

Efforts to shift power were described as not straight-forward and contradictory at times, for example where LSHTM staff asked partners to lead something, but partners wanted LSHTM to do it. LSHTM participants described sharing authorship where partners did not want to be co-authors. The tensions of shifting power where capacity was limited was also discussed as challenging. LSHTM actors said they felt pressured to mobilise “peer researchers” or engage refugees in data collection in settings where that was not possible, but also felt they were not being as collaborative as they could be by failing to use peer researchers. Although LSHTM actors were quite critical of their own efforts to shift power, interestingly partners felt more positively about these efforts, indicating that LSHTM actors encouraged them to use their expertise and emphasised that every actor had something to contribute. Some partners felt more comfortable in challenging LSHTM: “Partners do feel comfortable pushing back: I am more verbal… I vocalise what I don’t like” (Interview 13, woman, partner, LSHTM).

#### Internal dynamics within partner organisations.

LSHTM and partner participants reflected on the dynamics within partner organisations that shape research visits. The key issue was seniority (including how it intersects with other hierarchies).

Seniority was described as playing a significant role in shaping interactions between partner staff: “So we have some power dynamics because we normally, we are not happy to bypass our senior person” (Interview 19, man, mid/senior, partner). Traditional academic expectations and structures influenced these dynamics. More junior partner staff were described as not receiving acknowledgement for their work: “I’ve worked with partner organisations where you know, the more junior people do all the work, and the senior person swoops in and takes all the glory, and then doesn’t want the junior people’s names to be on reflected on, you know, papers or reports” (Interview 12, woman, mid-career, LSHTM). During one feedback session, LSHTM actors also shared experiences witnessing bullying of junior partner staff by senior partner staff. LSHTM actors reflected on partner institutions being a reflection of societal hierarchies: “I think African societies are still very hierarchical… If the boss says something you don’t contradict it… you also don’t question it” (Interview 14, woman, senior, LSHTM). These dynamics within partner organisations meant it was challenging when LSHTM actors tried to shift power hierarchies:

“If you’re a senior, if you’re a [Principal Investigator] or a professor, the senior researcher… there’s a clear chain of command. Things are not flattened… then we come … ‘Oh, no, no, we want to share power’, and they’re probably going, ‘What?’ Because that’s not how their teams are running” (Interview 12, woman, mid-career, LSHTM).

However, there were also examples of partner universities being more intentional in flattening hierarchies as part of a broader strategy to have more equitable research collaborations.

Seniority intersected with other dynamics including gender and economic status. One participant reflected: “The people in more senior positions were male and from like a relatively comfortable socioeconomic background. We did have some collaborators who are female, but they tended to have more of the lower-level jobs… They very rarely contributed in terms of discussions about the work. So I never really got to know them very well… because the work was presented by a more senior male colleague” (Interview 15, woman, early-career, LSHTM). Participants reflected on how seniority and gender were often difficult to disentangle. Gendered hierarchies at times resulted in negative dynamics within some partner organisations, including “domineering” and “authoritarian” behaviour by men and female staff being treated differently to male staff (Interview 9, man, early-career, LSHTM).

There was also discussion of how power hierarchies within partner organisations could even be more powerful than those within LSHTM: “I think, as a generalisation, that the strength of the power dynamics probably are much stronger within our partner organisations” (Interview 18, man, senior, LSHTM). Partner staff were recognised as often representing an elite, with higher access to education and higher class and economic status than the communities they were working with.

#### Dynamics with research participants and other stakeholders.

In this final section we discuss dynamics with research participants and other stakeholders, exploring how the idea of the ‘field’ reflects a particular power hierarchy with participants, how LSHTM actors being perceived as externals/outsiders affects the research dynamic and the challenges of engaging with stakeholders who hold power such as government actors.

**Concept of ‘the field’:** Some participants questioned the concept of ‘the field’:

“If I’ve come to your village, I’m not in the field. I’m not coming to take soil samples. I’m coming to ask you or observe you in your life, and I’m an outsider… [Saying field] dehumanises people and also homogenises people…” (Interview 7, woman, senior, LSHTM).

LSHTM participants also reflected on how partners reacted negatively about “the field” being used to describe where they worked or lived. In one feedback session, a partner commented: “It's hugely dehumanising to look at communities as a field, like it is somewhere where you test something”. There was some discussion of alternative terminology, but also recognition that “the field” was often used colloquially and for ease even if people fundamentally disagreed with the concept.

**External/outsider researcher perceptions:** LSHTM and partners discussed the specific power dynamics involved in engaging with research participants. Engaging directly with participants was perceived as creating very specific hierarchies: “That’s like massive power dynamics, you know, being the only person who shows up in a vehicle, and a 4-wheel drive” (Interview 2, woman, senior, LSHTM). LSHTM actors reflected on their role, recognising that it was not always possible or the best approach for LSHTM actors to engage with participants: “Someone like me going there and doing data collection or being involved with direct data collection could be a hindrance” (Interview 3, man, early-career, LSHTM). LSHTM participants described their own experiences collecting data and recognising the negative impact their presence had on research participants because of these dynamics.

There were some differences in opinion about LSHTM’s role in “observing” data collection, for example observing focus group discussions being conducted, with some feeling like this was a distraction and others, including partners, indicating it was important for LSHTM actors to themselves see and experience the challenges of data collection. One LSHTM actor shared how a local researcher pushed back against another LSHTM actor coming to observe saying: “No, she’s not just coming to watch, I don’t know this person… it’s not like a free for all” (Interview 1, woman, early-career, LSHTM). LSHTM actors, particularly students, described the challenges of responding to individual participant requests for help, including support with English and reviewing CVs. They did not always know how to respond to personal questions from research participants and did not always feel equipped to respond to sensitive research participant disclosures. LSHTM participants also reflected on the pressure to engage their research participants in co-design, reflecting that despite this push towards this ideal, practical and capacity considerations made this impossible.

### Challenges engaging with external stakeholders

Engaging with other stakeholders, including government actors and non-governmental organisations (NGOs) was perhaps the most challenging for LSHTM actors. These gatekeepers sometimes had high expectations for supporting researchers, including the expectation that they would be paid: “The first comment was often, ‘Well, what are you paying me for this interview?’… And I reflected on that after like, maybe I should have been offering some kind of small stipend” (Interview 6, woman, early-career, LSHTM).

Not being senior enough was often discussed as a barrier when engaging gatekeepers. For example, one participant discussed how her nationality and age worked against her when trying to interview government staff within a department “which is extremely hierarchical, extremely ageist”. She felt “they really wanted to see someone in a suit” (Interview 10, woman, early-career, LSHTM). Another staff member waited multiple days to meet an official, and felt she had to behave in a particular way to get their support: “I just remember I felt very much like a supplicant in that process…I wanted to be humble and respectful, and kind of understanding (Interview 6, woman, early-career, LSHTM). Senior staff also reflected that it was easier to interview government actors when they were perceived as senior: “I definitely when I was more junior, had been very ignored by more senior people in government” (Interview 18, man, senior, LSHTM). Importantly, race or being an outsider made it easier to access senior officials:

“[G]etting to meet people from like even the highest level of authority within their organization and knowing I sort of had that privilege also made me very uncomfortable… I felt like I was getting yeah, kind of special privileges for really no reason at all other than being a foreigner” (Interview 8, woman, early-career, LSHTM).

Gender also complicated power hierarchies, and sometimes required women “to do the gendered thing being asked to show respect” (Interview 2, woman, senior, LSHTM). Race and gender could sometimes be played off each other, for example, needing a man’s help could be emphasised to reduce the impact of being white:

“Maybe I’ll lean a little bit into the kind of male female power dynamic of like. Yes, you’re a big man. Yes, I’m willing to sort of acknowledge that. And I’m here asking you for your expertise, and I think in a way. I kind of probably leaned a little bit that way, in hopes maybe of kind of reducing the race barrier in a way. Yes, I’m white, but I’m also a woman, and therefore, you know, I’m very much acknowledging your expertise” (Interview 6, woman, early-career, LSHTM).

These hierarchies with other stakeholders could create complications for LSHTM actors, who may be dependent on gatekeepers who were behaving poorly: “She kind of started shouting at the peer researchers… They were so nice to me because I was a foreigner, and they wanted to take pictures with me. But then she was shouting at everyone else” (Interview 1, woman, early-career, LSHTM).

## Discussion

This paper contributes important insights into complex, often-intersecting power hierarchies that are present at multiple levels during research interactions between those in the North and South, highlighting tensions and contradictions researchers may encounter in their efforts to shift power. This case study of practices within an academic institution in the United Kingdom provides broader insights about complex power hierarchies operating within academia, with implications for how research collaborations are structured, how knowledge/expertise of actors in the North versus the South are positioned, and the role of institutional power hierarchies in shaping interpersonal relations [53].

We find that power itself is not always easy to understand as a concept, and more work is needed to help researchers from different disciplines intentionally reflect on how power hierarchies (and their intersections) impact research and are time- and context-dependent. Power hierarchies are best understood during “moments” in the research process rather than being positioned as static [44]. Deeper reflexivity about intersecting power hierarchies may involve “discomfort” [54–56]. This discomfort was perhaps reflected in the uncertainty LSHTM actors expressed about their future role in research, as well as in views, particularly among senior staff, that power dynamics are not always harmful or that privilege can be leveraged to empower others. Unease about perceptions that research trips have become difficult to navigate because of power dynamics, is also part of this discomfort. Framing research travel as routine may feel easier than engaging in intentional reflection, care and a change to established research practices. More work may be needed to what role Northern institutions can or should play in the long-term especially as funders shift towards directly funding organisations in the South instead of going through Northern actors [34,45].

The intersecting power hierarchies described in the findings illustrate that unequal power dynamics have real impacts on research interactions. Across multiple relationships, it is clear that seniority operates as a powerful dynamic within the field of research, shaped by wider hierarchies within academia that privilege senior researchers. The perception among early-career researchers that senior researchers are less motivated and interested in addressing unequal power dynamics is important and highlights the key tension that senior researchers have more to lose from shifts in power during research and that the push for more equitable, decolonised research may mean they have to relinquish power. The recognition among some participants of gender hierarchies (especially in LSHTM engagement with external stakeholders) is important, however it is unclear if there is consistent awareness of how gender affects dynamics. It may be that within LSHTM the superseding power of seniority dominates research dynamics, causing less attention to be placed on gender, however it is important to recognise that gendered power hierarchies remain a pervasive challenge within academia [57–59].

Aligning with the perspective that power hierarchies constantly shift and that power is not solely fixed in the North [9,44–46], this study also identified that hierarchical structures *within* partner organisations, particularly related to the intersection of seniority and gender, can cause harm, limiting the opportunities of partner staff who are women. Importantly, in many settings, these hierarchies were inherited from Western systems, including during colonialism [60]. Additionally, the power used by external stakeholders is an area where more support may be needed for LSHTM actors and partners to navigate these dynamics.

Although this study did not specifically seek to explore how institutional practices shape dynamics, it is clear from both LSHTM actors and partner staff that the significant power of LSHTM within research collaborations has an impact. Burdensome institutional practices regarding contracting, insurance and accounting are not unique to this institution [12], however in our study were identified as thwarting individual efforts to shift power. LSHTM actors’ frustration that their efforts were rendered ineffective against the budget power wielded by LSHTM highlights this is an important structural barrier that requires action. This study identifies key areas where institutional power hierarchies shape interactions between LSHTM actors and partners, including in ways that cause harm. Most identified by partners was the issue of arbitrary and Northern-centered decision-making about scheduling and the scope of visits. Importantly, other examples of problematic behaviour also emerged, which may be tied to institutional power but also other intersectional power dynamics including seniority.

Echoing other studies, we find that efforts to shift power to Global South actors are not always straight-forward [61,62]. Even if there is motivation to change dynamics among Northern researchers, more work is needed to ensure partners who have historically been subject to the power of institutions like LSHTM are invested in and prepared for new power-sharing approaches. More work is needed to ensure efforts to shift power, e.g., through providing authorship, are relevant and desired by partners, instead of the benefits being measured in Northern terms [61,63].

Finally, this study draws attention to the problems with the concept of the term “field”, which can create a dichotomy between “us” versus “them” and reinforce a clinical and extractive way of thinking about research that is grounded in the colonial underpinnings of this concept of the “field” [34]. Research participants in this study identified how the framing of the “field” is tied to the role of researchers in producing knowledge, reflecting on how the terminology that is used can reinforce top-down and Other-ising mindsets. Efforts to challenge this kind of thinking can draw on feminist and other ways of challenging what and whose knowledge counts [2,64], as well as more relational approaches to research that prioritise people over data, such as “ethics of care” approaches [65,66].

This study has some limitations. Firstly, this study represents only a single snapshot of power dynamics at the time of data collection. Recognising power dynamics and relationships are constantly shifting, a deeper analysis would involve longitudinal data collection. Secondly, it is impossible to fully understand all the intersecting power hierarchies that impact research processes. We have tried to present analyses that reflect this complexity as much as possible, but acknowledge that power operates both implicitly and explicitly and some interactions are difficult to identify. Thirdly, the recruitment approach of LSHTM actors might have resulted in individuals with more negative experiences of power hierarchies wanting to participate. This risk was slightly reduced by direct invitations to researchers involved in partnerships to join interviews. Fourth, it was challenging to identify senior and mid-career LSHTM staff members to participate, which might have meant the perspectives of early-career researchers are slightly more present in the findings. Fifth, some partner staff may have not felt completely comfortable in sharing their perspectives. Effort was made to ensure participants understood how their data would be used and the importance of confidentiality and anonymity, and this did result in some partners being very open about negative experiences, but it is possible that there was a concern among others that sharing their experiences would affect potential or current partnerships. It is worth noting that multiple LSHTM and partner participants shared examples that have not been included in this paper at their request, because these would identify them.

The following recommendations emerge from this study, focusing both on practical actions as well as recommended future research on power hierarchies. These recommendations are primarily for Northern institutions engaged in research collaborations, but may also have applicability for actors based in the South:

1. Challenge extractive practices and underpinning assumptions that are often associated with research in the “field”. This should go deeper than merely using replacement terminology to drawing attention to processes that reinforce an “us versus them” way of engaging with research participants and external stakeholders. Approaches like ethics of care in research may be useful to counter hierarchical and harmful engagement with those in the Global South [65,66].
2. Advocate for equitable institutional policies and practices related to contracting, insurance and other administrative and financial issues within Northern institutions, because these inevitably shape power dynamics within research collaborations. The efforts of individual researchers to identify and tackle unequal power hierarchies are important, but efforts to meaningfully shift power are often constrained by institutional policies and practices.
3. Set aside time for ongoing reflection on power and positionality within the research team throughout the project, guided by tools like ‘The space between us’ and the ‘Power flower tool’ [67,68] and focused on actionable steps that can be taken to challenge power hierarchies. Ongoing reflection is important to recognise that power dynamics constantly shift and are fluid. This reflection requires honest, and sometimes uncomfortable, conversations about the role of seniority as an overarching dynamic that often affects research processes, and identification of practical opportunities for early-career and more junior researchers to engage in decision-making and leadership. Such conversations should include discussing the purpose of any planned visits and who is best placed to travel. Reflections about North-South dynamics should involve open conversations about what shifting power looks like not just from the view of Northern actors, but grounded in what is desired by and helpful for those in the South. Discussions about power should go beyond research collaborations and should also be encouraged *within* institutions – not just between institutions.
4. Ensure the agenda and scheduling of visits is planned intentionally, at the convenience of Global South partners, instead of being imposed based on what suits those in the “North”. This means recognising that especially when funds are held by those in the “North” it creates a power dynamic that makes it more challenging for partners to push back on requests to visit, so intentional efforts need to be made.
5. Conduct further research on power hierarchies to identify other strategies to tackle specific dimensions of power. This includes research on how best to support early career researchers in sharing their concerns about problematic power dynamics and behaviours, while recognising the career impact of reporting these through institutional mechanisms. Research is needed to explore how senior researchers perceive power hierarchies and efforts to challenge power, recognising senior researchers often have the most to lose from efforts to decolonise research or incorporate equitable research practices. More work is needed to understand how best to ensure senior researchers are not left behind within conversations about shifting power that seem to be occurring more commonly among early-career and junior researchers, and what the role of research institutions like LSHTM might look like in the future. Research is also needed to better understand hierarchies *within* Global South organisations, recognising that power dynamics related especially to seniority and gender may have a powerful influence in these organisations. There is a need for more complex understanding of power as not solely North-South. More research and guidance on navigating dynamics with external stakeholders, such as government actors, is also needed to help researchers.

Alongside these recommendations, we emphasise the importance of broader use of well-established strategies to shift power throughout the research process – not just during visits. This may include using participatory approaches to design research grounded in expressed needs, challenging assumptions about what counts as knowledge, sharing leadership of the grant, being upfront about the budget, building in power-sharing governance approaches in the study, identifying joint values and ways of working, identifying each partner’s capacity, establishing collaboration agreements, agreeing on joint expectations for behaviour, ensuring appropriate reporting mechanisms exist for both Northern and Southern actors to report bullying/harassment/violence that occurs during research, establishing authorship plans, and budgeting for more informal social activities among the research team [1,6,7,10,11,12,21,24-30,36,61]. The use of these strategies should be based on collective decisions among the team, rather than a list of imposed actions, to ensure partners are comfortable with these changes to typical research collaborations.

## Conclusion

This case study of an academic institution with a colonial legacy in the UK provides important insights about intersecting power hierarchies that are present within research interactions in the “field”. Although discussing power inequities within research has become more common in recent years, our study suggests more work is needed for researchers to understand how power hierarchies impact their interactions with others, and how power is not static but evolves over time and based on context. We find that positionality shapes how participants view the role of power hierarchies in research, with senior staff tending to be less critical of power hierarchies, compared to early-career researchers. Our study identifies seniority as a key dimension that shapes interactions between researchers. Institutional power also constrains individual researcher efforts to shift power. Our findings raise broader questions about the role of Northern-based academic institutions within a climate increasingly focused on shifting power to the Global South and directly funding Southern institutions. However, we also identify that in the short-term, there is much that Northern-based academic institutions could do to ensure respectful and healthy interactions.

## Supporting information

S1 File Author positionality. (DOCX)
