# Supplementary material for: Navigating power hierarchies in the “field”: A qualitative exploration of researcher experiences
Source: PLOS Glob Public Health. 2026 Mar 11;6(3):e0006048. doi: 10.1371/journal.pgph.0006048 (PMC12978487; doi:10.1371/journal.pgph.0006048)
Supplement: S1 File — (DOCX) [file pgph.0006048.s001.docx]

**Supplementary file**

Author positionality

Michelle Lokot is a woman of colour, of Sri Lankan ethnicity and Australian nationality. She has lived in post-colonial contexts such as Brunei and Australia and now resides in the United Kingdom. Her perspectives on power and decolonisation have been shaped by reflections on her own role in perpetuating colonial dynamics while working as a humanitarian practitioner in multiple settings. She has been involved in the Equitable Partnerships Coordinating Group and Decolonising Global Health initiative at LSHTM.

Agata Pacho is a white European migrant, sociologist, and early-career researcher working in health research in the UK. She has been involved in the Decolonising Global Health initiative at the London School of Hygiene and Tropical Medicine (LSHTM). Her work and insights draw on sociological perspectives as well as learning from abolitionist movements.

Thurayya Zreik is a Lebanese early-career researcher who has spent most of her life and career in the Arab world. Her perspectives on power, knowledge hierarchies, and research practices have been shaped by her experience navigating dynamics in institutions and organizations in Western, traditionally colonial contexts as well as in post-colonial contexts in the Arab region. She has experience working and consulting for research and humanitarian institutions in both the "Global North" and "Global South", which has allowed her to recognize the different ways power dynamics can shape decision-making and participation in both institutional and humanitarian settings.

Rahma Hassan is a black African woman of Somali ethnicity and a Kenyan national. She grew up in rural Kenya and has worked over the years with marginalized and minority groups, such as pastoralists, where she comes from. Her perspectives on power stem from her own experiences working in global teams as a researcher, recognizing the different layers of privilege that education and positions present, which create distinct dynamics in collaborations. This includes the imbalance in access to resources for research and knowledge sharing that privileges those who can pay and research practices that value outputs done by experts and not stories told by local people. She has been involved in reflecting on power dynamics in co-producing research as well as supporting organisations addressing systemic challenges as well as enhancing equitable partnerships.

Nada Abdelmagid is a Muslim, Arabic-speaking Black African woman born and raised in Sudan, currently living and working in the United Kingdom in health research.  Prior to working in academia in the UK, she had almost a decade of experience at sub-national, national, and international levels in the humanitarian health sector within its traditional architecture. Nada has both personal and vicarious experiences of boundary crossing, witnessing manifestations of power and colonial dynamics in postcolonial contexts and Western centres of power.

Mishal Khan is a Muslim woman of colour born in Pakistan and raised in the UAE. She has insider-outsider experience of post-colonial contexts from her childhood and her education in elite British universities.  Mishal regularly speaks about narrow and prejudiced ideas about who holds expertise. Her research focuses on improving health systems and policies in South and Southeast Asia and she has worked in Pakistan, Singapore, and the UK. She is currently a Professor of Global Public Health and governing body member of a UK school of public health, where she was involved in an Independent Review to address discrimination and advance anti-racism and equality in 2021. She also holds a visiting faculty position at the Aga Khan University in Karachi, Pakistan and is a member of The Lancet Group for Racial Equality.
